# The role of antipsychotics and other drugs on the development and progression of neuroleptic malignant syndrome

**DOI:** 10.1038/s41598-023-45783-z

**Published:** 2023-10-27

**Authors:** Yoji Kyotani, Jing Zhao, Kiichi Nakahira, Masanori Yoshizumi

**Affiliations:** Department of Pharmacology, Nara Medical University School of Medicine, 840 Shijo-Cho, Kashihara, Nara 634-8521 Japan

**Keywords:** Adverse effects, Combination drug therapy, Schizophrenia

## Abstract

Neuroleptic malignant syndrome (NMS) is a rare but serious and sometimes fatal complication in patients taking antipsychotic drugs, and its underlying mechanism still remains unclear. The pharmacotherapy for psychotic disorders is complicated and often involves a combination of two or more drugs, including drugs other than antipsychotics. In the present study, we used the Japanese Adverse Drug Event Report (JADER) database to broadly investigate the drugs associated with NMS, following their related pathways, as well as the drug-drug interactions (DDIs) in NMS. All analyses were performed using data from the JADER database from April 2004 to May 2022. Single-drug signals were evaluated using the reporting odds ratio (ROR) and proportional reporting ratio (PRR), and drug pathways were investigated using the Kyoto Encyclopedia of Genes and Genomes (KEGG). DDIs were evaluated using the Ω shrinkage measure and Chi-square statistics models. All drugs associated with 20 or more NMS cases in the JADER database exhibited signals for NMS, including non-antipsychotics. Pathways associated with the drugs included the dopaminergic or serotonergic synapses related to antipsychotics. DDIs leading to NMS were confirmed for several drug combinations exhibiting single-drug signals. This study confirmed the significant association of various drugs, including non-psychotics, with NMS and suggested that various pathways related to these drugs may be involved in the progression of NMS. In addition, several combinations of these drugs were found to interact (DDI), increasing the risk of NMS, which suggests that appropriate caution should be taken when administering these drugs.

## Introduction

Neuroleptic malignant syndrome (NMS) is well known as a rare but serious complication of antipsychotic drugs and is characterized by the following symptoms; fever, muscle rigidity, hyperpyrexia, autonomic dysfunction, mental disorders and abnormal metabolic changes^1,2^. Without an early diagnosis and appropriate treatment, NMS can be fatal, particularly when accompanied by respiratory changes, severe hyperthermia, and older age^3^. The etiology and pathophysiology of NMS remain unclear; however, have been suggested to involve the sudden depletion of central dopamine due to antipsychotic-induced D2-receptor antagonism^1,2^. Moreover, NMS has also been reported as an adverse event (AE) following the use of non-antidopaminergic medications^1,4–6^, and many drugs, including non-antipsychotic drugs used to treat psychiatric diseases, may play a role in NMS development.

The World Health Organization has defined pharmacovigilance as science and activities related to the detection, assessment, understanding, and prevention of adverse effects or any other medicine/vaccine-related problem. In pharmacovigilance, spontaneous reporting systems (SRS), such as the Japanese Adverse Drug Event Report (JADER) database and the U.S. Food and Drug Administration's Adverse Event Reporting System (FAERS), are used to detect signals of previously unknown or incomplete information regarding a possible causal relationship between an AE and a drug. The reporting odds ratio (ROR) and proportional reporting ratio (PRR) were developed to detect drug-related AEs using disproportionality analysis. In addition, Ω shrinkage measures and Chi-square statistics models have also been proposed to detect drug-drug interactions (DDIs, defined as an increase in the effect of one drug due to the presence of another drug) in AEs.

This study aimed to clarify the drugs and pathways that may be involved in NMS, along with DDIs that may increase the risk of NMS. Therefore, we extensively investigated the association between drugs and NMS, as well as DDIs leading to NMS, using the JADER database.

## Materials and methods

### Data sources

The JADER database collects spontaneous AE reports in Japan, similar to the FAERS database, and is available on the Pharmaceuticals and Medical Devices Agency (PMDA) website (www.pmda.go.jp). The JADER database complies with the International Safety Reporting Guidelines (ICH E2B) and consists of four data files represented as data tables: 1) demo.csv (patient demographic information); 2) drug.csv (drug information); 3) reac.csv (AE information); and 4) hist.csv (underlying disease). The drug file contains the role code assigned to each drug: suspected, interacting, or concomitant. Data from April 2004 to May 2022 were downloaded from the JADER database, and records with missing values in the drug name, role code, start date, and adverse event date columns were removed. The drug and AE information tables were combined using the ID as the key and all drugs registered as suspected in the role code were analyzed. On the other hand, FAERS data from the 1st quarter of 2018 to the 4th quarter of 2022 were downloaded from the U.S. Food and Drug Administration website (www.fda.gov). The FAERS database consists of seven data files named ‘DEMO’, ‘DRUG’, ‘REAC’, ‘OUTC’, ‘RPSR’, ‘THER’, and ‘INDI’. In analyses, data of the ‘DEMO’, ‘DRUG’, and ‘REAC’ files were combined using the ID, and limited to physician (MD) for the reporter’s occupation and primary and secondary suspect drugs (PS and SS) for drug’s role in the event. In addition, we followed the FDA recommendation to adopt the most recent CASE number to identify duplicate reports of the same patient from different reporting sources and excluded them from the analysis.

The pathways for each drug were obtained from the Kyoto Encyclopedia of Genes and Genomes (KEGG) database (https://www.kegg.jp/kegg/).

### Definition of AEs and drug names

The target AE in this study was NMS (preferred term (PT) code:10,029,282), which was extracted from the data table based on PTs from the Medical Dictionary for Regulation Activities, Japanese version 25.1 (MedDRA/J v25.1). Drug names were referred to using the International Non-proprietary Name (INN) or the United States Adopted Name (USAN), whereas drugs that were not described by INN or USAN were referred to using the Japanese Pharmacopoeia, 18^th^ edition (JP18).

### Statistical methods

Contingency tables (2 × 2 and 4 × 2) were created for signal detection and analyzed using the appropriate methods (Table 1 and 2). As the use of multiple methods is recommended for optimal signal detection, the ROR and PRR methods were applied for the single-drug signal, and the Ω shrinkage measure and Chi-square statistics models were applied for DDIs^7^. The single-drug signal was examined for drugs reported in at least 20 cases of NMS. DDIs were examined, with a statistically significant signal for each drug in the ROR or PRR and at least three NMS reports for the respective drug combination. To validate our results, FAERS was used to examine the ROR and PRR for ingredients associated with drugs for which a single drug signal was detected in JADER, and the ingredients with a statistically significant signal and at least 15 cases of NMS were examined for DDIs.

**Table 1:** 2 × 2 contingency table for ROR and PRR.

	NMS	All other AEs	Total
Target drug	N_11_	N_10_	N_1+_
All other drugs	N_01_	N_00_	N_0+_
Total	N_+1_	N_+0_	N_++_

*AEs* adverse events, *NMS* neuroleptic malignant syndrome, *PRR* proportional reporting ratios, *ROR* reporting odds ratio.

**Table 2:** 4 × 2 contingency table for Ω shrinkage measure and Chi-square statistics.

	NMS	All other AEs	Total
Target drug D_1_ and D_2_	n_111_	n_110_	n_11+_
Only drug D_1_	n_101_	n_100_	n_10+_
Only drug D_2_	n_011_	n_010_	n_01+_
Neither drug D_1_ nor drug D_2_	n_001_	n_000_	n_00+_
Total	n_++1_	n_++0_	n_+++_

*AEs* adverse events, *NMS* neuroleptic malignant syndrome.

### Reporting odds ratio (ROR)

The ROR is a signal detection index used by the Netherlands Pharmacovigilance Centre Lareb^7^. The ROR and 95% confidence intervals (CIs) of NMS caused by the target drug were calculated using data from the reports shown in Table 1 and Eqs. (1) and (2). To be defined as a signal, the lower limit of the 95% CI of the ROR had to be greater than 1^7,8^.

1 $$ROR= \frac{{N}_{11}/{N}_{10}}{{N}_{01}/{N}_{00}}$$

2 $$ROR\, \left(95\%\, CI\right)= {e}^{\mathrm{ln}(ROR) \pm 1.96\sqrt{\frac{1}{{N}_{11}}+ \frac{1}{{N}_{10}}+\frac{1}{{N}_{01}}+\frac{1}{{N}_{00}}}}$$

### Proportional reporting ratio (PRR)

PRR is a signal detection index used by the European Medicines Agency^7^. The PRR and Chi-squared values were calculated using data from the reports shown in Table 1 and Eqs. (3), (4), and (5). The PRR signal criteria were PRR ≥ 2, Chi-square value ≥ 4, and N_11_ ≥ 3^7,9,10^.

3 $$PRR= \frac{{N}_{11}/{N}_{1+}}{{N}_{01}/{N}_{0+}}$$

4 $$PRR\, \left(95\% \,CI\right)= {e}^{\mathrm{ln}(PRR) \pm 1.96\sqrt{\frac{1}{{N}_{11}}+ \frac{1}{{N}_{10}}+\frac{1}{{N}_{01}}+\frac{1}{{N}_{00}}}}$$

5 $${\chi }^{2}= \frac{{N}_{++}{\left(\left|{N}_{11}{N}_{00}-{N}_{10}{N}_{01}\right|-{N}_{++}/2\right)}^{2}}{{N}_{1+}{N}_{+1}{N}_{0+}{N}_{+0}}$$

### The Ω shrinkage measure model

The Ω shrinkage measure is the model used for DDI detection that returns the most conservative results^11,12^. The shrinkage measure proposed by Norén et al^12^. is shown below as Eq. (6),

6 $$\Omega = {\mathrm{log}}_{2}\frac{{n}_{111}+\alpha }{{E}_{111}+\alpha }$$

where E_111_ is the expected value for the incidence of NMS suspected to be derived from the DDI, and α is a tuning parameter to determine shrinkage strength and provides enough shrinkage to avoid the highlighting of disproportional reporting based on just one or two reports when α equals 0.5. From a Bayesian perspective, Ω can be viewed as the logarithm of the posterior mean of the unknown rate of incidence. Since the posterior distribution of incidence μ will be gamma distribution due to conjugacy, the choice of the prior is made purely for mathematical convenience. With the Bayesian approach, exact credibility interval limits for μ can be calculated using Eq. (7), for appropriate posterior quantiles μ_q_.

7 $${\int }_{0}^{{\mu }_{q}}\frac{{\left({E}_{111}+\alpha \right)}^{{n}_{111}+\alpha }}{\Gamma \left({n}_{111}+\alpha \right)}{u}^{{n}_{111}+\alpha -1}{e}^{-\left({E}_{111}+\alpha \right)u}du=q$$

The logarithm of the solution to Eq. (7) for q = 0.025 provides the lower limit of a two-sided 95% credibility interval for Ω (Ω_025_). The DDI signal was calculated for Ω_025_ > 0, because Ω_025_ > 0 is used as a threshold to screen for signals under the concomitant use of two drugs^12,13^.

### Chi-square statistics model

Chi-square statistics is a relatively new model for screening DDIs proposed by Gosho et al.^14^ and demonstrates a higher sensitivity than that of the Ω shrinkage measure model when events are rare^14^. The detection criterion for DDIs was set to χ > 2 as calculated using Eq. (8).

8 $$\chi = \frac{{n}_{111}-{E}_{111}-0.5}{\sqrt{{E}_{111}}}$$

## Results

### Signal detection for the association between drugs and NMS

A total of 2991 NMS cases were identified in the JADER database. Approximately 36 drugs were reported in 20 or more NMS cases, and signals were detected in both the ROR and PRR (Table 3). Of these drugs, “perospirone hydrochloride hydrate” and “chlorpromazine hydrochloride, promethazine hydrochloride, and phenobarbital” are only sold in Japan, although the latter has been off the market since 2017. In addition, whether ingredients associated with the drugs detected the signals in JADER were investigated the ROR and PRR for NMS in FAERS, all of them were found to be the statistically significant (Supplementary Table S1).

**Table 3:** PRRs and RORs for drugs with NMS reported in JADER.

Drug	Cases	Non-cases	Total	χ^2^	PRR (95% CI)	ROR (95% CI)
Total	2991	1,121,146	1,124,137			
Risperidone	252	2282	2534	8929.07	40.72 (35.57–46.63)	45.11 (39.40–51.65)
Aripiprazole	220	2282	2502	6838.47	35.59 (30.84–41.08)	38.93 (33.73–44.92)
Haloperidol	206	796	1002	15,486.77	82.91 (70.82–97.07)	104.11 (88.92–121.89)
Olanzapine	151	1805	1956	4074.30	30.50 (25.73–36.16)	32.97 (27.82–39.08)
Quetiapine fumarate	121	1716	1837	2746.43	25.76 (21.34–31.08)	27.50 (22.79–33.19)
Blonanserin	118	644	762	6598.68	60.55 (49.59–73.93)	71.46 (58.53–87.25)
Paroxetine hydrochloride hemihydrate	71	2853	2924	508.31	9.32 (7.35–11.83)	9.53 (7.51–12.10)
Levomepromazine maleate	71	575	646	2761.32	42.29 (32.96–54.26)	47.39 (36.93–60.80)
Biperiden hydrochloride	62	435	497	2747.04	47.86 (36.59–62.60)	54.54 (41.69–71.33)
Perospirone hydrochloride hydrate*	58	316	374	3218.14	59.42 (44.80–78.80)	70.14 (52.89–93.02)
Sulpiride	58	929	987	1150.68	22.50 (17.22–29.41)	23.85 (18.24–31.17)
Lithium carbonate	56	806	862	1238.55	24.86 (18.92–32.68)	26.52 (20.18–34.86)
Flunitrazepam	56	980	1036	1012.83	20.68 (15.76–27.14)	21.81 (16.62–28.62)
Amantadine hydrochloride	53	801	854	1114.09	23.73 (17.93–31.41)	25.23 (19.06–33.40)
Paliperidone	40	414	454	1217.58	33.55 (24.20–46.50)	36.69 (26.47–50.86)
Donepezil hydrochloride	39	1720	1759	245.42	8.43 (6.12–11.60)	8.60 (6.25–11.83)
Carbamazepine	34	5083	5117	29.25	2.51 (1.79–3.53)	2.52 (1.80–3.54)
Chlorpromazine hydrochloride	33	292	325	1160.77	38.58 (26.87–55.39)	42.82 (29.82–61.49)
Fluvoxamine maleate	31	837	868	345.29	13.55 (9.45–19.43)	14.02 (9.78–20.10)
Brexpiprazole	30	185	215	1467.03	52.96 (35.95–78.03)	61.39 (41.67–90.44)
Midazolam	30	1073	1103	241.34	10.32 (7.16–14.85)	10.58 (7.34–15.23)
Milnacipran hydrochloride	30	392	422	719.36	26.98 (18.58–39.18)	28.97 (19.95–42.06)
Etizolam	29	964	993	253.97	11.07 (7.64–16.05)	11.38 (7.85–16.49)
Tiapride hydrochloride	28	280	308	871.20	34.48 (23.34–50.94)	37.83 (25.61–55.88)
Promethazine hydrochloride	27	164	191	1333.13	53.60 (35.62–80.66)	62.26 (41.38–93.70)
Zotepine	27	257	284	879.66	36.05 (24.21–53.68)	39.73 (26.68–59.16)
Clozapine	27	2204	2231	71.57	4.58 (3.13–6.71)	4.62 (3.16–6.77)
Paliperidone palmitate	27	643	670	343.83	15.27 (10.38–22.49)	15.87 (10.78–23.37)
Clomipramine hydrochloride	26	222	248	937.80	39.74 (26.43–59.75)	44.28 (29.45–66.57)
Valproate sodium	26	2469	2495	53.85	3.94 (2.67–5.81)	3.97 (2.70–5.86)
Chlorpromazine hydrochloride, promethazine hydrochloride and phenobarbital (1)*^,†^ (Suspension of sale)	25	273	298	710.91	31.79 (21.07–47.95)	34.61 (22.94–52.21)
Asenapine maleate	25	257	282	753.94	33.59 (22.24–50.73)	36.76 (24.34–55.52)
Sertraline hydrochloride	23	1200	1223	114.26	7.12 (4.70–10.77)	7.23 (4.78–10.94)
Amoxapine	23	384	407	424.86	21.40 (14.03–32.64)	22.62 (14.83–34.50)
Levodopa and benserazide hydrochloride	20	234	254	525.84	29.79 (18.84–47.09)	32.25 (20.40–50.98)
Haloperidol decanoate	20	90	110	1263.99	68.79 (42.32–111.82)	83.85 (51.58–136.31)

*NMS* neuroleptic malignant syndrome, *PRR* proportional reporting ratios, *ROR* reporting odds ratio.

*Sold only in Japan.

^†^The numbers are assigned because they were different product names due to differences in standards.

### Pathways in signal-detected drugs

**Figure 1:**
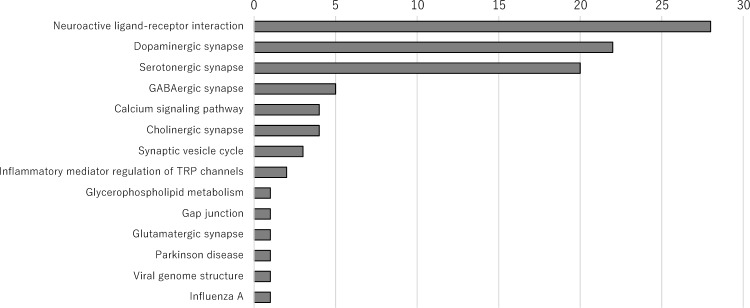
Pathways of Drugs with Statistically Significant RORs and PRRs. The frequencies of pathways associated with drugs with statistically significant ROR and PRR values are summarized. The number for each pathway represents the number of the pathway in all the target drugs *PRR* proportional reporting ratio; *ROR* reporting odds ratio; *TRP* transient receptor potential.

We used the KEGG database to identify the pathways associated with signal-detected drugs (Fig. 1, Supplementary Table S2 and S3)^15^. The lithium carbonate pathways were not included in Fig. 1 because lithium carbonate does not have a KEGG pathway, and the pathways for medical compounds containing several drugs were referenced for each drug. The following detected pathways are listed according to their frequency of occurrence: neuroactive ligand-receptor interaction (hsa04080), dopaminergic synapse (hsa04728), serotonergic synapse (hsa04726), GABAergic synapse (hsa04727), calcium signaling pathway (hsa04020), cholinergic synapse (hsa04725), synaptic vesicle cycle (hsa04721), inflammatory mediator regulation of transient receptor potential channels (hsa04750), glycerophospholipid metabolism (hsa00564), gap junction (hsa04540), glutamatergic synapse (hsa04724), Parkinson’s disease (hsa05012), viral genome structure (ko03230), and influenza A (ko05164). As expected, most of the pathways related to NMS were neuroactive ligand-receptor interactions, and dopaminergic and serotonergic synapses.

### Drug-drug interactions in NMS

To investigate DDIs leading to NMS, we performed the Ω shrinkage measure and Chi-square statistics models for all drugs that exhibited ROR and PRR signals (Supplementary Data). We summarized the signals detected using the Ω shrinkage measure or Chi-square statistics models for all drug combinations listed in Table 3 (Table 4). All signals detected by the Ω shrinkage measure model were confirmed using the Chi-square statistics model (Table 4 and Supplementary Data). On the other hand, of the ingredients for which the signals for NMS were detected in Supplementary Table S1, the DDIs for all drugs with 15 or more NMS case reports were also investigated and revealed that combinations of the ingredients showed the DDIs in FAERS were not necessarily the same as those in JADER (Supplementary Table S4).

**Table 4:** Detected signals of NMS due to concomitant drug use.

Combination of drugs (ROR of single drug)		${n}_{111}$	${E}_{111}$	${\Omega }_{025}$	$\chi$
Risperidone (45.11)	Lithium carbonate (26.52)	15	6.62	0.30	3.06
	Clomipramine hydrochloride (44.28)	6	1.48	0.34	3.30
Aripiprazole (38.93)	Olanzapine (32.97)	23	13.29	0.12	2.53
	Milnacipran hydrochloride (28.97)	3	0.70	− 0.50	2.16
Haloperidol (104.11)	Lithium carbonate (26.52)	6	1.90	0.06	2.61
Olanzapine (32.97)	Chlorpromazine hydrochloride, promethazine hydrochloride and phenobarbital (1)*^,†^ (Suspension of sale) (34.61)	6	1.96	0.03	2.53
	Sertraline hydrochloride (7.23)	5	1.72	− 0.22	2.12
Paroxetine hydrochloride hemihydrate (9.53)	Amantadine hydrochloride (25.23)	3	0.70	− 0.51	2.15
Lithium carbonate (26.52)	Etizolam (11.38)	3	0.50	− 0.24	2.84
	Zotepine (39.73)	10	1.75	1.19	5.86
	Chlorpromazine hydrochloride, promethazine hydrochloride and phenobarbital (1)*^,†^ (Suspension of sale) (34.61)	5	1.69	− 0.20	2.16
Donepezil hydrochloride (8.60)	Carbamazepine (2.52)	3	0.65	− 0.45	2.29
	Fluvoxamine maleate (14.02)	3	0.70	− 0.50	2.16
Carbamazepine (2.52)	Fluvoxamine maleate (14.02)	3	0.69	− 0.49	2.18
Fluvoxamine maleate (14.02)	Clomipramine hydrochloride (44.28)	7	1.55	0.61	3.98
Etizolam (11.38)	Zotepine (39.73)	4	0.61	0.28	3.69

*NMS* neuroleptic malignant syndrome, *ROR* reporting odds ratio.

*Sold only in Japan.

^†^The numbers are assigned because they were different product names due to differences in standards.

## Discussion

NMS, which is an important cause of morbidity and mortality in patients taking antipsychotics, has been reported not only in patients taking antipsychotics, but also in those taking non-psychotics, and its underlying mechanisms still remain unclear^2,4–6^. Therefore, we used the JADER database to investigate the association between NMS and single-drug use, including non-antipsychotics, as well as its association with concomitant drug use. As a result of our analyses, we drew the following inferences in the present study: (1) various drugs, including antipsychotics, anti-Parkinson’s, anti-anxiety, and antiepileptic drugs, were suggested to be associated with NMS; (2) the NMS-related pathways inferred by signal detection were mainly neuroactive ligand-receptor interactions and dopaminergic and serotonergic synapses, although other pathways may also be involved; and (3) several combinations of drugs were found to interact (DDI), increasing the risk of NMS development.

In the single-drug signal analysis, all drugs with 20 or more NMS case reports in JADER demonstrated signals for NMS (Table 3). Although there are a number of previous papers reporting associations between drugs and NMS, only the most relevant drugs are included in Table 3^4–6,16–18^. In addition, the present study included drugs for which there are few or no previous reports of an association with NMS. That is, the drugs that exhibited signals were mainly antipsychotics but also included non-antipsychotics. Although detected signals, especially for drugs with few or no previous reports of NMS, do not necessarily indicate a causal relationship between the drug and NMS, they do indicate a sufficient likelihood of validation^7^. Pathways associated with the components of the drug, except lithium carbonate, were mainly neuroactive ligand-receptor interactions, dopaminergic synapses, or serotonergic synapses; however, other pathways that were not associated with dopamine or serotonin were also identified (Fig. 1). Since the pathways identified in this study are based on the drugs for which signals were detected, these pathways are not necessarily implicated in NMS. However, of the pathways shown in Fig. 1, most frequent pathways were of psychotropic drugs which have NMS as an adverse event, such as dopaminergic and serotonergic synapses. There are also reports of NMS resulting from discontinuation or switching of dopamine agonists for Parkinson's disease^19^. In fact, one of the frequent pathways was dopaminergic synapse, and its association with the next most frequent serotonergic synapse is well known^20^. GABA inhibits dopaminergic activity^21–23^, and dopamine promotes calcium signaling via multiple mechanisms^24,25^. The pathway of cholinergic synapse includes drugs used to treat Parkinson's and Alzheimer's disease, and psychotic symptoms in Alzheimer's disease are treated by atypical antipsychotics, suggesting the involvement of cholinergic synapse in dopamine pathway. The pathway shown in Fig. 1 includes many pathways that involve dopamine, and may support the idea that the rapid depletion of dopamine stimulation leads to the development of NMS.

It has been reported that abrupt discontinuation or rapid switching of dopaminergic drugs for Parkinson’s disease^19^ may precipitate NMS due to the abrupt withdrawal of D2 receptor stimulation. On the other hand, “amantadine hydrochloride” and “levodopa and benserazide hydrochloride”, which are antiparkinsonism drugs and provide dopamine to the central nervous system, are listed as suspected drugs for NMS in this study (Table 3). Taking into account that the abrupt withdrawal of D2 receptor stimulation may be involved in the development of NMS, it is presumed that NMS occurred upon withdrawal or tapering of these drugs.

A previous study suggested an association between genetic defects and NMS, reporting that a mother and her two daughters presented with NMS^26^. If genetic defects are in fact associated with the development of NMS, genes or proteins related to the pathways shown in Fig. 1 may be involved. However, all previous reports on NMS are either case reports or SRS analyses limited by drug category, primary disease, and/or other variables^3–6,16,17,27^. Therefore, the inference in this study is more general and may provide clues for elucidating the developmental mechanism of NMS.

The proportion of AEs attributed to DDIs is estimated to be between 6 and 30%, and the analysis of the safety profile of DDIs is challenging but important^28^. In the present study, we applied the Ω shrinkage measure and Chi-square statistics models to all drugs identified during single-drug signal analysis to detect DDIs that may lead to NMS, and found that several drug combinations may have synergistic interactions leading to the development of NMS, as shown in Table 4. Several case reports have suggested DDIs between clomipramine/risperidone, aripiprazole/olanzapine, or lithium/risperidone, and a pharmacovigilance study compared the occurrence of NMS following monotherapy and combination therapy with atypical antipsychotics and haloperidol monotherapy using the JADER database^27,29–34^. The DDIs reported in previous case reports on NMS were confirmed by both the Ω shrinkage measure and Chi-square statistics models in the present study, supporting the idea that some drug combinations may synergistically result in NMS. However, the results obtained in this study differ significantly from those of previous reports in that they are more general. Specifically, the drugs that elicit DDIs in NMS were not necessarily typical antipsychotic drugs but also included atypical antipsychotic, anti-Parkinson’s, anti-anxiety, and antiepileptic drugs, as demonstrated by the Chi-square statistics model (Table 4). Among the DDIs in Table 4, the concomitant use of risperidone and lithium is one of the most common in previous case reports of NMS. Risperidone is a relatively potent dopamine receptor blocker compared with other atypical antipsychotics. On the other hand, it has been reported that chronic lithium treatment in rodents limits the increase of extracellular dopamine in the nucleus accumbens and prevents intracellular signaling via the dopamine D2 receptor^35^. These facts also seem to support the idea that the rapid depletion of dopamine stimulation leads to the development of NMS. Thus, the risk of NMS with risperidone may be increased by the concomitant use of lithium.

Although the Ω shrinkage measure and Chi-square statistics models demonstrate a similar detection tendency for DDIs, the latter is more sensitive when events are rare^11,14^. Therefore, by taking the results from both models into account, it is possible that various pathways, mainly neuroactive ligand-receptor interactions, and dopaminergic, or serotonergic synapses, may interact with each other and lead to the development of NMS. It is important to note that confounding factors due to co-medication or underlying disease could not be excluded in the investigation of DDIs because the number of cases for each combination of drugs was small and further stratified analysis for DDI detection would be difficult^12,14^. However, our results still suggest that caution should be taken by healthcare professionals when administering these drugs.

Because the JADER database is based on SRS, the data are subject to certain limitations, such as under-reporting of AEs, variable quality of reports, and reporting bias^36^. Normally, it is impossible to analyze SRS data to quantify the extent of risk; however, statistically detected signals can suggest a hypothesis that should be substantiated or possibly confirmed by clinical trials or observational studies. If a potential safety signal is detected, pharmacoepidemiological studies may be required to obtain information on the risk or protective factors. Furthermore, since the pathways in Fig. 1 are based on signals detected using the JADER database, pharmacoepidemiological studies may be needed as well as for drugs for which a signal is detected.

The FAERS was used to validate the results of the single drug signals and DDIs using JADER in this study. Although the single drug signals using FAERS were detected in all of the ingredients associated with the drugs detected the signals in JADER, the DDIs for the concomitant use of the ingredients were significantly different between JADER and FAERS. Previous reports have shown that differences between databases can arise as a result of discrepancies in reporting rules and customs which are rooted in regulations^37,38^. Therefore, the differences in the results of the DDIs can be attributed to the differences between the JADER and FAERS. On the other hand, the results of the drug signals suggest that the ingredients of the drugs may be causal for NMS regardless of the databases.

Overall, few studies have comprehensively examined the relationship between drugs and NMS. In addition, there have been no comprehensive studies of DDIs in NMS. We believe that this study provides new insights into these issues.

## Conclusions

The present study broadly confirmed the association between several drugs and NMS, and identified their pathways. Overarching observations suggest that pathways other than the well-known dopaminergic or serotonergic synapses may also be involved in the development of NMS. Furthermore, DDIs leading to NMS were confirmed for several drug combinations that exhibited single-drug signals, suggesting that the concomitant use of these drugs may increase the risk of NMS. Thus, extra caution should be exercised when administering these drug combinations.

### Supplementary Information

Supplementary Information.

Supplementary Tables.

## Data Availability

All data generated in this study are included in this published article and its supplementary information files. The data set of the JADER database can be downloaded from the Japanese website of the Pharmaceuticals and Medical Devices Agency (https://www.pmda.go.jp), but it is in Japanese only.
