# Supplementary material for: The role of antipsychotics and other drugs on the development and progression of neuroleptic malignant syndrome
Source: Sci Rep. 2023 Oct 27;13:18459. doi: 10.1038/s41598-023-45783-z (PMC10611799; doi:10.1038/s41598-023-45783-z)
Supplement: Supplementary file 2 — Supplementary Tables. [file 41598_2023_45783_MOESM2_ESM.docx]

**The role of antipsychotics and other drugs on the development and progression of Neuroleptic Malignant Syndrome**

Yoji Kyotani^1,*^, Jing Zhao^1^, Kiichi Nakahira^1^, and Masanori Yoshizumi^1^

1. *Department of Pharmacology, Nara Medical University School of Medicine; 840 Shijo-cho, Kashihara, Nara 634-8521, Japan.*

^*^Corresponding author: Yoji Kyotani

E-mail address: [cd147@naramed-u.ac.jp](mailto:cd147@naramed-u.ac.jp)

ORCID: 0000-0002-7771-6889

Supplementary Table S1 PRRs and RORs for drugs in Table 1 with NMS reported in FAERS

| Drug | Cases | Non-cases | Total | χ^2^ | PRR (95% CI) | ROR (95% CI) |
| --- | --- | --- | --- | --- | --- | --- |
| Total | 2940 | 7940442 | 7943382 |  |  |  |
| Risperidone | 197 | 20245 | 20442 | 4731.89 | 27.84 (24.07-32.19) | 28.1 (24.3-32.49) |
| Aripiprazole | 153 | 22164 | 22317 | 2526.83 | 19.49 (16.55-22.94) | 19.61 (16.66-23.09) |
| Haloperidol | 186 | 8839 | 9025 | 9948.78 | 59.38 (51.11-68.98) | 60.6 (52.17-70.41) |
| Haloperidol Lactate | 2 | 115 | 117 | 49.02 | 46.22 (11.41-187.11) | 47 (11.61-190.3) |
| Olanzapine | 215 | 22011 | 22226 | 5188.74 | 28.12 (24.46-32.33) | 28.38 (24.69-32.63) |
| Olanzapine Pamoate | 3 | 905 | 908 | 13.94 | 8.93 (2.87-27.77) | 8.96 (2.88-27.85) |
| Quetiapine | 194 | 27657 | 27851 | 3268.25 | 20.08 (17.35-23.24) | 20.21 (17.47-23.39) |
| Quetiapine Fumarate | 68 | 12895 | 12963 | 821.08 | 14.48 (11.38-18.43) | 14.56 (11.44-18.52) |
| Blonanserin | 1 | 117 | 118 | 4.77 | 22.9 (3.2-164.02) | 23.09 (3.22-165.37) |
| Paroxetine | 21 | 6500 | 6521 | 135.70 | 8.76 (5.7-13.46) | 8.78 (5.71-13.5) |
| Paroxetine Hydrochloride | 8 | 3137 | 3145 | 34.51 | 6.89 (3.44-13.8) | 6.9 (3.45-13.83) |
| Paroxetine Mesylate | 1 | 50 | 51 | 12.27 | 52.99 (7.32-383.76) | 54.03 (7.46-391.29) |
| Levomepromazine | 17 | 1430 | 1447 | 476.14 | 31.92 (19.76-51.56) | 32.29 (19.99-52.16) |
| Levomepromazine Maleate | 1 | 96 | 97 | 6.00 | 27.86 (3.88-199.9) | 28.14 (3.92-201.91) |
| Biperiden Hydrochloride | 10 | 698 | 708 | 325.82 | 38.29 (20.49-71.55) | 38.82 (20.77-72.55) |
| Biperiden | 8 | 740 | 748 | 188.54 | 28.97 (14.42-58.21) | 29.28 (14.57-58.81) |
| Sulpiride | 5 | 650 | 655 | 74.81 | 20.66 (8.56-49.84) | 20.81 (8.63-50.2) |
| Lithium | 60 | 4966 | 5026 | 1787.79 | 32.91 (25.44-42.56) | 33.29 (25.74-43.05) |
| Lithium Carbonate | 27 | 3174 | 3201 | 541.34 | 22.99 (15.71-33.64) | 23.18 (15.84-33.91) |
| Lithium Citrate | 2 | 44 | 46 | 129.22 | 117.55 (28.48-485.12) | 122.85 (29.77-506.99) |
| Flunitrazepam | 5 | 401 | 406 | 125.96 | 33.33 (13.79-80.57) | 33.73 (13.95-81.55) |
| Amantadine Hydrochloride | 7 | 1091 | 1098 | 91.42 | 17.26 (8.2-36.33) | 17.37 (8.25-36.55) |
| Amantadine | 6 | 985 | 991 | 71.87 | 16.39 (7.34-36.6) | 16.48 (7.38-36.81) |
| Paliperidone | 19 | 2142 | 2161 | 391.96 | 23.9 (15.19-37.6) | 24.11 (15.32-37.92) |
| Donepezil | 10 | 2666 | 2676 | 73.16 | 10.13 (5.44-18.86) | 10.16 (5.46-18.93) |
| Donepezil Hydrochloride | 4 | 1322 | 1326 | 18.46 | 8.16 (3.06-21.79) | 8.18 (3.06-21.85) |
| Carbamazepine | 8 | 9666 | 9674 | 4.30 | 2.24 (1.12-4.48) | 2.24 (1.12-4.48) |
| Chlorpromazine | 23 | 2103 | 2126 | 599.54 | 29.45 (19.5-44.49) | 29.76 (19.7-44.96) |
| Chlorpromazine Hydrochloride | 7 | 377 | 384 | 284.53 | 49.37 (23.35-104.36) | 50.27 (23.78-106.26) |
| Fluvoxamine | 10 | 880 | 890 | 255.43 | 30.46 (16.31-56.87) | 30.79 (16.49-57.49) |
| Fluvoxamine Maleate | 2 | 572 | 574 | 7.81 | 9.42 (2.35-37.77) | 9.45 (2.36-37.89) |
| Brexpiprazole | 12 | 1311 | 1323 | 247.70 | 24.6 (13.92-43.48) | 24.82 (14.04-43.87) |
| Midazolam | 7 | 2981 | 2988 | 26.33 | 6.34 (3.02-13.33) | 6.35 (3.02-13.35) |
| Midazolam Hydrochloride | 4 | 2436 | 2440 | 7.47 | 4.43 (1.66-11.83) | 4.44 (1.66-11.85) |
| Etizolam | 2 | 458 | 460 | 10.39 | 11.75 (2.93-47.16) | 11.8 (2.94-47.35) |
| Tiapride | 7 | 302 | 309 | 356.69 | 61.35 (28.97-129.91) | 62.75 (29.63-132.87) |
| Tiapride Hydrochloride | 5 | 478 | 483 | 104.50 | 28.02 (11.6-67.67) | 28.3 (11.72-68.35) |
| Promethazine Hydrochloride | 35 | 2639 | 2674 | 1135.43 | 35.78 (25.58-50.04) | 36.24 (25.91-50.68) |
| Zotepine | 3 | 90 | 93 | 176.68 | 87.24 (27.6-275.78) | 90.12 (28.51-284.86) |
| Clozapine | 143 | 31993 | 32136 | 1440.50 | 12.59 (10.64-14.89) | 12.64 (10.68-14.96) |
| Paliperidone Palmitate | 26 | 3691 | 3717 | 423.39 | 19.06 (12.94-28.08) | 19.19 (13.02-28.26) |
| Clomipramine Hydrochloride | 7 | 927 | 934 | 109.62 | 20.3 (9.64-42.73) | 20.44 (9.71-43.04) |
| Clomipramine | 5 | 1266 | 1271 | 34.54 | 10.65 (4.42-25.64) | 10.68 (4.44-25.73) |
| Valproate Sodium | 18 | 7203 | 7221 | 82.37 | 6.77 (4.26-10.77) | 6.78 (4.27-10.79) |
| Asenapine Maleate | 8 | 945 | 953 | 144.90 | 22.74 (11.33-45.65) | 22.92 (11.42-46.02) |
| Sertraline Hydrochloride | 21 | 24259 | 24280 | 14.80 | 2.35 (1.53-3.61) | 2.35 (1.53-3.61) |
| Amoxapine | 2 | 44 | 46 | 129.22 | 117.55 (28.48-485.12) | 122.85 (29.77-506.99) |
| Benserazide;Levodopa | 2 | 711 | 713 | 5.79 | 7.58 (1.89-30.4) | 7.6 (1.9-30.47) |
| Haloperidol Decanoate | 6 | 660 | 666 | 112.02 | 24.39 (10.91-54.53) | 24.6 (11-55) |
| *NMS* neuroleptic malignant syndrome, *PRR* proportional reporting ratios, *ROR* reporting odds ratio | | | | | | |

Supplementary Table S2 KEGG pathways for each drug

| Drug | KEGG pathway |
| --- | --- |
| Risperidone | hsa04080, hsa04726, hsa04728 |
| Aripiprazole | hsa04080, hsa04726, hsa04728 |
| Haloperidol | hsa04080, hsa04728 |
| Olanzapine | hsa04080, hsa04726, hsa04728 |
| Quetiapine fumarate | hsa04080, hsa04726, hsa04728 |
| Blonanserin | hsa04080, hsa04726, hsa04728 |
| Paroxetine hydrochloride hemihydrate | hsa04726 |
| Levomepromazine maleate | hsa04080, hsa04728 |
| Biperiden hydrochloride | hsa04080, hsa04725 |
| Perospirone hydrochloride hydrate^*^ | hsa04020, hsa04080, hsa04540 |
| Sulpiride | hsa04080, hsa04728 |
| Lithium carbonate |  |
| Flunitrazepam | hsa04080, hsa04727 |
| Amantadine hydrochloride | hsa04080, hsa04724, hsa04728, ko03230, ko05164 |
| Paliperidone | hsa04080, hsa04726, hsa04728 |
| Donepezil hydrochloride | hsa00564, hsa04725 |
| Carbamazepine | hsa04728 |
| Chlorpromazine hydrochloride | hsa04080, hsa04725, hsa04726, hsa04728 |
| Fluvoxamine maleate | hsa04726 |
| Brexpiprazole | hsa04080, hsa04726, hsa04728 |
| Midazolam | hsa04080, hsa04727 |
| Milnacipran hydrochloride | hsa04721, hsa04726 |
| Etizolam | hsa04080, hsa04727 |
| Tiapride hydrochloride | hsa04080, hsa04728 |
| Promethazine hydrochloride | hsa04020, hsa04080, hsa04750 |
| Zotepine | hsa04080, hsa04726, hsa04728 |
| Clozapine | hsa04080, hsa04726, hsa04728 |
| Paliperidone palmitate | hsa04080, hsa04726, hsa04728 |
| Clomipramine hydrochloride | hsa04721, hsa04726 |
| Valproate sodium | hsa04020, hsa04727 |
| Chlorpromazine hydrochloride, promethazine hydrochloride and phenobarbital^*^ (Suspension of sale) | hsa04080, hsa04725, hsa04726, hsa04728; hsa04020, hsa04080, hsa04750; hsa04080, hsa04727 |
| Asenapine maleate | hsa04080, hsa04726, hsa04728 |
| Sertraline hydrochloride | hsa04726 |
| Amoxapine | hsa04721, hsa04726 |
| Levodopa and benserazide hydrochloride | hso04020, hsa04080, hsa04728, hsa05012; hsa04726, hsa04728 |
| Haloperidol decanoate | hsa04080, hsa04728 |
| ^*^ sold only in Japan | |

Supplementary Table S3 Correspondence of KEGG pathway and pathway map

| KEGG pathway | KEGG pathway map |
| --- | --- |
| hsa00230 | Purine metabolism |
| hsa00564 | Glycerophospholipid metabolism |
| hsa00590 | Arachidonic acid metabolism |
| hsa00670 | One carbon pool by folate |
| hsa00790 | Folate biosynthesis |
| hsa02010 | ABC transporters |
| hsa04010 | MAPK signaling pathway |
| hsa04020 | Calcium signaling pathway |
| hsa04060 | Cytokine-cytokine receptor interaction |
| hsa04064 | NF-kappa B signaling pathway |
| hsa04080 | Neuroactive ligand-receptor interaction |
| hsa04146 | Peroxisome |
| hsa04210 | Apoptosis |
| hsa04218 | Cellular senescence |
| hsa04260 | Cardiac muscle contraction |
| hsa04261 | Adrenergic signaling in cardiomyocytes |
| hsa04270 | Vascular smooth muscle contraction |
| hsa04350 | TGF-beta signaling pathway |
| hsa04380 | Osteoclast differentiation |
| hsa04512 | ECM-receptor interaction |
| hsa04540 | Gap junction |
| hsa04611 | Platelet activation |
| hsa04612 | Antigen processing and presentation |
| hsa04614 | Renin-angiotensin system |
| hsa04650 | Natural killer cell mediated cytotoxicity |
| hsa04658 | Th1 and Th2 cell differentiation |
| hsa04659 | Th17 cell differentiation |
| hsa04660 | T cell receptor signaling pathway |
| hsa04721 | Synaptic vesicle cycle |
| hsa04724 | Glutamatergic synapse |
| hsa04725 | Cholinergic synapse |
| hsa04726 | Serotonergic synapse |
| hsa04727 | GABAergic synapse |
| hsa04728 | Dopaminergic synapse |
| hsa04750 | Inflammatory mediator regulation of TRP channels |
| hsa04911 | Insulin secretion |
| hsa04918 | Thyroid hormone synthesis |
| hsa04920 | Adipocytokine signaling pathway |
| hsa04930 | Type II diabetes mellitus |
| hsa04960 | Aldosterone-regulated sodium reabsorption |
| hsa04966 | Collecting duct acid secretion |
| hsa04971 | Gastric acid secretion |
| hsa05012 | Parkinson disease |
| hsa05321 | Inflammatory bowel disease |
| hsa05323 | Rheumatoid arthritis |
| ko00100 | Steroid biosynthesis |
| ko00550 | Peptidoglycan biosynthesis |
| ko00790 | Folate biosynthesis |
| ko03010 | Ribosome |
| ko03020 | RNA polymerase |
| ko03230 | Viral genome structure |
| ko05164 | Influenza A |

Supplementary Table S4 Detected signals of NMS due to concomitant drug use in FAERS

| Combination of drugs (ROR of single drug) | | $n_{111}$ | $E_{111}$ | $\Omega_{025}$ | $\chi$ |
| --- | --- | --- | --- | --- | --- |
| Aripiprazole (19.61) | Lithium Carbonate (23.18) | 12 | 5.50 | 0.13 | 2.56 |
| Haloperidol (60.6) | Quetiapine (20.21) | 50 | 37.01 | 9.61E-16 | 2.05 |
| Paroxetine (8.78) | Clozapine (12.64) | 5 | 0.62 | 0.77 | 4.94 |
| Promethazine Hydrochloride (36.24) | Olanzapine (28.38) | 8 | 1.87 | 0.67 | 4.11 |
|  | Lithium (33.29) | 14 | 1.49 | 2.01 | 9.84 |
|  | Lithium Carbonate (23.18) | 6 | 1.09 | 0.65 | 4.22 |
|  | Chlorpromazine (29.76) | 6 | 1.09 | 0.65 | 4.22 |
|  | Sertraline Hydrochloride (2.35) | 5 | 1.51 | -0.08 | 2.44 |
| *NMS* neuroleptic malignant syndrome, *ROR* reporting odds ratio | | | | | |
